# Rituximab for autoimmune blistering diseases in pregnancy and children: a clinical review

**DOI:** 10.1097/JW9.0000000000000270

**Published:** 2026-06-25

**Authors:** Henry Tseng, John W. Frew, Dédée F. Murrell

**Affiliations:** a Department of Dermatology, Liverpool Hospital, Sydney, New South Wales, Australia; b School of Clinical Medicine, Faculty of Medicine and Health, UNSW Sydney, New South Wales, Australia; c Department of Dermatology, St. George Hospital, Sydney, New South Wales, Australia

**Keywords:** autoimmune blistering diseases, fetal exposure to drugs, pediatrics, pemphigus, pregnancy complications, rituximab

## Abstract

**Objective:**

To critically review the current literature on the safety, efficacy, and clinical considerations of rituximab use in pediatric and pregnant patients with autoimmune blistering diseases (AIBD).

**Data Sources:**

A comprehensive literature search was conducted using PubMed and Embase to identify relevant articles, including case reports, case series, observational studies, and systematic reviews.

**Study Selections:**

Studies were selected based on relevance to rituximab use in pediatric and antenatal AIBD, with particular focus on treatment efficacy, adverse outcomes, and long-term immunologic effects.

**Results:**

Rituximab demonstrates promising efficacy in pediatric AIBD, though concerns remain regarding persistent hypogammaglobulinemia and delayed immune reconstitution. In regards to pregnant patients, current data do not suggest a consistent association between rituximab exposure and congenital anomalies or serious neonatal complications, particularly when used preconception or early in gestation. Reported neonatal B-cell depletion has been transient. Multidisciplinary oversight is critical given the clinical complexity of these populations.

**Conclusion:**

Rituximab may be a viable corticosteroid-sparing option for severe or refractory AIBD in children and pregnant women. Its use should be individualized and carefully monitored by experienced multidisciplinary teams. Larger prospective studies are needed to inform evidence-based guidelines.

**What is known about this subject in regard to women and their families?**

- Autoimmune blistering diseases (AIBDs) such as pemphigus and pemphigoid can present during pregnancy or childhood, posing significant diagnostic and therapeutic challenges.
- Pregnant women with AIBD face limited treatment options because of safety concerns for both mother and fetus.
- Rituximab is a monoclonal antibody increasingly used in severe or refractory AIBD, but its use during pregnancy is off-label, with safety data largely derived from case reports and small cohorts.

**What is new from this article as messages for women and their families?**

- Current evidence suggests that rituximab exposure before conception or in early pregnancy is not associated with a consistent pattern of birth defects or serious neonatal complications.
- Neonatal B-cell depletion following in-utero rituximab exposure is generally transient, with immune recovery typically occurring within 6 months.
- This review highlights emerging evidence supporting rituximab as a potential corticosteroid-sparing option in select high-risk pregnancies and underscores the importance of multidisciplinary care. Individualized risk–benefit discussions, guided by experienced, multidisciplinary clinicians, are essential when considering rituximab during pregnancy.

## Introduction

Autoimmune blistering diseases (AIBDs) are a clinically heterogeneous group of rare, life-threatening disorders that present with autoantibody-mediated blistering of the skin and mucosae.^1^ Of these, pemphigus vulgaris (PV) and bullous pemphigoid are the most common with distinct immunopathogenic characteristics. The management of AIBDs has historically relied upon systemic corticosteroids and broad-spectrum immunosuppressants.^1^ These therapies are, though, often followed by serious morbidity, particularly among high-risk patient populations like children and pregnant women, in whom the dangers of growth retardation, infection, teratogenicity, and obstetric morbidity pose significant therapeutic dilemmas.

Rituximab, a chimeric monoclonal antibody against the CD20 antigen on B cells, has been a game changer in the therapy for AIBDs, primarily PV. Landmark trials, such as the Ritux 3 trial, have established rituximab as an induction agent for first-line treatment of adult patients with moderate-to-severe pemphigus with improved long-term disease control and reduced corticosteroid exposure.^2^ This paradigm has witnessed increasing off-label use of rituximab in children and pregnant patients with refractory disease. The safety, efficacy, and long-term outcomes of rituximab in these 2 patient groups are currently inadequately characterized because of exclusion from major clinical trials and the rare prevalence of AIBDs in these populations.^3^

In the pediatric population, AIBDs are uncommon but may be severe, recalcitrant, and psychologically debilitating. Therapy is also compounded by the side effects of chronic immunosuppression during critical stages of development and growth.^4^ Rituximab has been found to be beneficial in small case series and retrospective reviews, particularly for pemphigus and refractory linear immunoglobulin A (IgA) disease, but evidence remains mixed. Similarly, during pregnancy, rituximab has been utilized off-label to control severe or chronic AIBD when conventional treatments are not effective or contraindicated. Transplacental transfer of rituximab is of concern for fetal B-cell depletion and neonatal immune suppression, but early evidence suggests that these effects generally do not lead to infectious complications if proper and careful application is ensured.^5^

With increasing reliance on rituximab in the management of complex AIBDs in such special populations, it is imperative for a critical synthesis of the currently available evidence. This review critically examines rituximab therapy in AIBDs in children and pregnant women based on clinical indications, treatment success, safety profiles, and practice guidelines. Our aim is to summarize current knowledge, to fill knowledge gaps in the literature, and to recommend evidence-based practice in such high-risk groups.

## Rituximab in pediatric AIBD

Pediatric AIBDs are uncommon, representing a small fraction of AIBDs worldwide. Despite their low incidence, their clinical significance is disproportionate because of diagnostic challenges, scarcity of therapeutic options, and the substantial psychosocial impact of childhood chronic disease. Pediatric AIBDs constitute a diverse group and consist of PV, juvenile bullous pemphigoid (JBP), linear IgA bullous dermatosis (also known as chronic bullous disease of childhood), and epidermolysis bullosa acquisita, with characteristic pathophysiologic and clinical features.

Childhood-onset PV, although rare, mirrors the adult form in the pathogenic process by desmoglein-targeted autoantibodies.^6,7^ The prognosis of pediatric pemphigus is considered to be better than that of adults, but is considerably variable.^8–10^ JBP typically presents in neonates or young children and may exhibit a less fulminant disease course than adult bullous pemphigoid, but severe or treatment-resistant disease can occur.^11,12^ Linear IgA bullous dermatosis is the most frequently seen bullous condition in children, with tense vesicles and characteristic “cluster of jewels” morphology, often being misdiagnosed at presentation.^13,14^ Epidermolysis bullosa acquisita, though rare in children, poses specific therapeutic challenges because of its resistance to conventional immunosuppression and potential for chronic scarring.^15^

The rarity of these conditions in children mirrors a paucity of pediatric-specific treatment protocols or randomized controlled trials.^16^ Treatment is thus typically extrapolated from adult protocols or anecdotal practice, with high heterogeneity of care and outcome. In these conditions, use of targeted biologics such as rituximab offers a logical, potentially paradigm-shifting treatment option. Several biologic agents have been used or considered off-label in pediatric AIBDs (Table 1).

**Table 1.** Biologics Used or Considered in Pediatric Autoimmune Blistering Diseases

Biologic	Off-label indications in pediatric AIBDs	Minimum approved age (any indication)	Evidence level	Safety profile in pediatrics
Rituximab	Refractory pemphigus vulgaris (approved for adults), bullous pemphigoid	≥2 y^17^	Moderate (case series, retrospective studies)	Generally well tolerated; risk of infusion reactions, infections
Omalizumab	Bullous pemphigoid	≥6 y^18^	Low (case reports)	Favorable; minimal adverse effects reported
Infliximab	Linear IgA disease, epidermolysis bullosa acquisita	≥6 y^19^	Low (case reports)	Risk of infections, infusion reactions
Dupilumab	Bullous pemphigoid (approved for adults)	≥6 mo^20^	Very low (isolated reports)	Favorable safety profile
IVIG	Pemphigus vulgaris, bullous pemphigoid	All ages	Moderate (case series, expert consensus)	Safe; transient headache, flu-like symptoms

AIBD, autoimmune blistering disease; IVIG, intravenous immunoglobulin; mo, month; y, year.

### Rationale for rituximab use

Pediatric AIBD application of rituximab is largely driven by the need for long-term disease control in corticosteroid-resistant and conventional immunosuppressives-resistant cases such as azathioprine, mycophenolate mofetil, or dapsone. Pediatric AIBD patients are more prone to a chronic and episodic course of disease than adults and often need long-term immunomodulation.^21,22^ However, chronic systemic immunosuppression in children has unique risks like disrupted linear growth, pubertal delay, osteopenia, susceptibility to opportunistic infections, and neurocognitive effects.^4,23^

Rituximab’s ability to induce long-term remission with possibly reducing the cumulative corticosteroid dose, has made it a valuable option in the pediatric setting.^24^ Because of the lack of controlled trials, rituximab is currently not approved by the United States Food and Drug Administration (FDA) for the treatment of pediatric AIBD, though it is noteworthy that rituximab is FDA-approved for treatment of granulomatosis with polyangiitis and microscopic polyangiitis in children aged 2 and older.^17^ Case reports and small numbers of cases have shown encouraging outcomes in children with AIBD, demonstrating marked reductions in disease activity and corticosteroid requirements. Importantly, rituximab has been successful in allowing steroid tapering or discontinuation, an important clinician-relevant endpoint in the pediatric setting.^24^

Furthermore, rituximab’s typically mild short-term tolerability in other autoimmune diseases in children, such as pediatric lupus and autoimmune cytopenias, has contributed to fueling its growing off-label usage in pediatric dermatology.^25^ Though long-term immunosuppression, hypogammaglobulinemia, and risk of infection are areas of concern, these must be weighed against chronic steroid exposure and the morbidity of inadequately controlled illness.^26^ Importantly, given the off-label nature of rituximab use in pediatric AIBD, careful counseling and informed parental consent are essential before initiation. In certain cases—particularly those involving widespread or markedly progressive disease that is unresponsive to initial therapy—rituximab may be the most rational and evidence-based choice.

### Evidence summary

Given the lack of randomized controlled trials, increasing evidence presented as case reports, case series, and observational studies supports the clinical application of rituximab in the management of pediatric AIBDs.^16^ One such study is the recent systematic review conducted by Shrivastava et al.^16^ that summarized outcomes of 18 studies consisting of 46 pediatric and adolescent patients with PV treated with rituximab. The review included a diverse set of study designs—case reports, case series, retrospective cohorts, a prospective study, and an open-label pilot study. Notably, nearly all patients achieved complete or partial remission, and many of them remained in remission at the latest follow-up with few adverse effects.^16^ In addition, a retrospective case series of 5 pediatric patients in Turkey showed similar results, with complete remission off therapy in 3 patients and partial remission in 2—with no adverse events observed.^27^ These findings underscore rituximab’s high therapeutic potential even in treatment-refractory cases, while also highlighting the need for harmonized treatment protocols. Nevertheless, severe side effects may occur, as seen in 1 pediatric patient in a study by Kong et al.^4^, whose course was complicated by neutropenia and sepsis after receiving a higher dose of rituximab (2 doses of 750 mg/m^2^ 2 weeks apart) and taking concomitant immunosuppressants, including high-dose corticosteroids.

The treatment regimens reported in the literature remain heterogeneous. Some clinicians adopt a fixed-schedule approach based on lymphoma protocols (375 mg/m^2^ weekly for 4 weeks), while others employ the low-dose regimen (2 doses of 500 mg 2 weeks apart), sometimes modified for pediatric weight.^16^ In the absence of pediatric-specific recommendations, extrapolation from adults or pediatric use in other autoimmune diseases (eg, juvenile idiopathic arthritis, granulomatosis with polyangiitis) is common practice.^17^ However, treatment protocols vary in terms of the ideal rituximab dose and treatment frequency, and the need for maintenance infusions are not yet established. Some studies favoring fixed dosing schedules, while others—such as the retrospective study by Wennmann et al.^28^—support a symptom-oriented and B cell–guided approach. Though this paper does not specifically address rituximab in pediatric patients with AIBD, it notes that reconstitution kinetics and treatment response can vary significantly both between and within individuals, depending on the underlying condition and dosing strategy.^28^

Recent cohort studies further endorse rituximab’s potential in children with AIBD but note the necessity for formalized pediatric dosing algorithms. Jamois et al.^17^ noted that children might metabolize rituximab differently than adults and might need pharmacokinetic optimization on an individual basis. Overall, these findings highlight the clinical efficacy of rituximab in children but reflect the critical deficiencies in the standardization of dosing, long-term persistence of treatment, and relapse prevention mechanisms.

### Safety and monitoring

Known immunologic vulnerabilities of pediatric patients need to be monitored closely when administering rituximab. The clinically most significant side effect is hypogammaglobulinemia. Prevalence rates of 44 to 52% for rituximab-induced hypogammaglobulinemia have been reported by Labrosse et al.^26^ and Khojah et al.^29^ in children, with a well-documented association between reduced IgG and risk of severe infection. Such findings oblige the monitoring of baseline and serial immunoglobulins, particularly IgG, both before and after rituximab therapy. Immunoglobulin replacement therapy may be used in specific cases, particularly in patients with recurrent infections or persistently low IgG.^26,30^

Rituximab’s mechanism of action—B-cell depletion of CD20+ cells—raises questions regarding vaccine efficacy as well. Live vaccines are contraindicated during and for several months following rituximab therapy because of a potential absence of immunogenicity and risk of disseminated infection.^31^ Further, even inactivated vaccines can induce suboptimal responses when administered during B-cell depletion.^31^ Thus, pretreatment immunization is strongly advised with attention to completion of age-appropriate vaccinations—all pneumococcal, meningococcal, and influenza vaccines—before initiating rituximab.

B-cell reconstitution in pediatric patients is extremely variable but typically occurs 6 to 12 months after treatment, with a median of approximately 11.8 months as reported by Wennmann et al.^28^ This prolonged immunologic nadir demands ongoing monitoring for infection and may influence redosing or maintenance therapy decisions. Of note, long-term consequences of repeat B-cell depletion in children remain partially unknown, particularly regarding immune system development and autoantibody recurrence. Emerging data indicate that persistent IgG hypogammaglobulinemia may occur in a substantial subset of patients, with 1 study reporting a 26.7% incidence among 101 evaluable children.^26^ Significant risk factors included low baseline IgG and IgA levels before rituximab therapy.^26^ These findings underscore the need for long-term immunologic follow-up in pediatric patients treated with B-cell–depleting therapies. Prudent patient selection, individualized risk–benefit consideration, and close liaison with pediatric immunologists are essential to optimize results.

## Rituximab use in pregnancy in the context of AIBDs

The use of rituximab during pregnancy in patients with AIBDs is still a complicated and underexplored area in clinical care. Corticosteroids and immunosuppressive agents, like azathioprine, have traditionally been preferred for disease control during pregnancy.^32,33^ However, in treatment-refractory cases, especially that of PV, serious treatment dilemmas do arise.

### Clinical indications and maternal considerations

Active AIBDs during pregnancy are associated with obstetric complications, such as preterm labor, intrauterine growth restriction, and fetal loss.^34^ According to the FDA, rituximab is currently contraindicated in pregnant women within 1 year of conception and during lactation.^35^ For women of reproductive age, selecting a treatment plan should take into account both effective disease management and counseling on contraception, along with discussions about future pregnancy intentions.^36^ Rituximab, very rarely, is instituted as a rescue therapy for the most severe corticosteroid-refractory cases to try to control disease activity and enhance maternal and fetal outcomes.^37^ Management of flares during gestation is important to decrease the risk of adverse fetal events such as neonatal pemphigus.^37^ As an added benefit, rituximab may permit the discontinuation of immunosuppressive therapy prior to and during pregnancy, avoiding the risks of immunosuppressive medications to the mother and their prospective fetuses.^36^ Despite such reports, there is little published research to date that are robust and specific to pregnant women with AIBD treated with rituximab.

### Placental transfer and timing of exposure

Rituximab, being an IgG1 monoclonal antibody, has minimal placental transfer during the first trimester. The transfer is known to increase substantially after the 16th week of gestation when there are concerns of fetal B-cell depletion.^38^ Accidental conception shortly after rituximab administration may not confer significant fetal exposure if in the first trimester because of the delay in active IgG1 placental transfer.^5^ Nevertheless, B-cell depletion seen in neonates has been reported as transient in 2 case reports, and the levels typically return to normal within about 6 months after birth.^39^ Key pharmacologic properties, safety considerations, and clinical implications of rituximab use during pregnancy are summarized in Table 2.

**Table 2.** Rituximab in pregnancy: summary table

Parameter	Details
Mechanism of action	Anti-CD20 monoclonal antibody; depletes B cells by complement and apoptosis^40^
Half-life	~22 d (range: 18–32 d); prolonged in repeated dosing^41^
Pregnancy category	Former FDA Category C (risk cannot be ruled out); now assessed case-by-case^42^
Placental transfer	Minimal in first trimester; significant in second/third via FcRn-mediated transport^38,43^
Known safety data	Case reports and registries show no major teratogenicity; transient neonatal B-cell depletion reported^44^
Breastfeeding compatibility	Likely safe; minimal transfer into breast milk; infant absorption negligible due to protein degradation^45^
Vaccination considerations	Live vaccines contraindicated in exposed neonates^46^

FcRn, neonatal fragment crystallizable receptor; FDA, Food and Drug Administration.

### Evidence from case series and registries

The discussion concerning the safety of rituximab during pregnancy has primarily unfolded around case series and cross-sectional studies. Table 3 summarizes reported pregnancy outcomes in patients with AIBD exposed to rituximab, including timing of exposure, maternal disease status, and neonatal outcomes. For example, a published case series by Perrotta et al.^44^ of 21 women exposed to rituximab before pregnancy or during pregnancy did not report neonatal B-cell depletion. Three infants had major structural anomalies, 2 were small for gestational age, yet there was never a pattern of congenital defects or serious adverse outcomes observed.^44^

**Table 3.** Summary of reported pregnancy outcomes in patients with autoimmune blistering disease after rituximab exposure

References	Underlying condition	No. of pregnancies	Timing of rituximab exposure	Live births	Preterm births	Low-birth weight	Major congenital anomalies	Neonatal B-cell depletion	Infections/sepsis	Other adverse events	Maternal disease course
Dehghanimahmoudabadi et al.^35^	Pemphigus	19	Pre-/during pregnancy	17	3	4	0	Not measured	1 (Neonatal sepsis)	1 Hydronephrosis	5 Flares during, 5 after pregnancy
Vassallo et al.^47^	Pemphigus	3	During pregnancy	3	0	0	0	Not specified	0	None reported	Controlled
Lake et al.^36^	Pemphigus	3	Prepregnancy	3	0	0	0	Not specified	0	None reported	1 Flare after pregnancy

Similarly, in a retrospective series of 21 pregnancies of patients with multiple sclerosis exposed to rituximab, no major fetal malformations were detected.^48^ A cross-sectional study by Dehghanimahmoudabadi et al.^35^ in pemphigus patients reported 19 pregnancies exposed either before or during pregnancy. Among 17 live births, 3 were preterm, and 4 were low birth weight; notably, 1 neonate experienced early-onset sepsis but recovered fully.^35^ Disease activity was fairly well maintained, with a few minor and major relapses during the pregnancy and postpartum period. Exposure to rituximab within 6 months of conception was associated with low-birth weights, possibly confounded by simultaneous corticosteroid use or underlying disease activity.^35^ The authors advised the avoidance of rituximab within 1 year of planned conception until more data are available.^35^ By contrast, favorable outcomes were reported in a small series of patients with pemphigus treated with rituximab during pregnancy by Vassallo et al.^47^ thus supporting its cautious use in refractory cases.

Though not investigating the AIBD population, another larger systematic review involving 102 pregnancies exposed to rituximab for multiple sclerosis or neuromyelitis optica spectrum disorder within 6 months of conception was reassuring with regard to major safety signals.^5^ Transient neonatal B-cell lymphopenia was detected in 39% of the newborns, with all infants demonstrating immune reconstitution within 6 months, and no maternal relapses occurring during pregnancy.^5^ One patient did have a postpartum relapse.^5^

### Neonatal outcomes and monitoring

Rituximab administration *in utero* can cause transient neonatal B-cell lymphopenia, with a theoretical possibility of increased infections or alteration in vaccine response during infancy. Although no consistent evidence suggests increased infection risk, monitoring neonatal B-cell counts via flow cytometry, particularly before live vaccinations, is recommended.^39^ Waiting to administer live vaccines until B-cell recovery is generally recommended.^49^

### Guidelines and recommendations

Given the ethical and logistical constraints of conducting randomized controlled trials in pregnant women, management of AIBDs during pregnancy has largely been founded on clinical experience and limited observational evidence. A key priority is the establishment of larger disease-specific registries to gain systematic data on maternal and neonatal outcomes and inform evidence-based recommendations. While case series suggest that rituximab may be an effective steroid-sparing agent in refractory disease, its use in pregnancy should be reserved for well-selected cases and managed on high-risk multidisciplinary teams accustomed to complicated obstetrics.

## Conclusion

Rituximab is a valuable option for the management of severe or refractory AIBD in childhood and pregnancy, particularly when first-line therapies are not adequate. Although its efficacy is well-established, long-term safety, especially concerning immune maturation and neonatal outcome, remains unclear. Its use needs to be monitored by skilled multidisciplinary teams, and larger prospective trials need to yield data regarding population-specific recommendations.

## Conflicts of interest

None.

## Funding

The Australasian Blistering Diseases Foundation (ABDF) provided funding to cover the open-access publication fees.

## Study approval

As this is a narrative review article, no ethics approval or institutional review board oversight was required.

## Author contributions

HT: Wrote the original manuscript. JWF and DFM: Made a substantial contribution by revising the manuscript critically related to the accuracy or integrity of any part of the work. All authors contributed to the article and approved the submitted version.
